# Rethinking the evidence for intensive surveillance after renal tumor ablation

**DOI:** 10.1007/s00330-026-12368-z

**Published:** 2026-02-17

**Authors:** Lisa C. Adams, Keno K. Bressem

**Affiliations:** Institute of Diagnostic and Interventional Radiology, TUM University Hospital, TUM School of Medicine and Health, Munich, Germany

We image because we are uncertain, and we are uncertain because we image. This circular logic underpins much of oncological surveillance, yet rarely do we pause to examine whether our vigilance serves patients or merely our own discomfort with inaction. In this issue of *European Radiology*, Reijerink and colleagues confront this question directly, presenting a systematic review of CT surveillance practices following thermal ablation for clinical T1 renal cell carcinoma (RCC) [1]. Their findings should unsettle anyone who assumes that current practice rests on solid evidence.

Among 37 studies encompassing over 6000 tumors, nearly 90% employed CT imaging frequencies exceeding the 2016 European Association of Urology (EAU) guideline recommendations, and all surpassed the 2024 guidelines. The data revealed something counterintuitive: recurrence rates were numerically lower in studies performing more frequent imaging (7.7%) compared to guideline-adherent studies (12.3%), though this difference was not statistically significant (*p* = 0.19). Before concluding that intensive surveillance therefore works, consider the alternative explanation: this likely reflects confounding, selection bias, and the fundamental limitation that only four studies adhered to guidelines, precluding meaningful comparison. The mean 5-year overall survival was 82.9% regardless of surveillance intensity, with cancer-specific survival exceeding 95%. What this review demonstrates is not that intensive imaging improves outcomes, but that we lack any evidence it does, despite two decades of practice predicated on that assumption.

This absence of supporting data exposes a striking asymmetry in how we evaluate surveillance practices. When introducing a new intervention, we demand randomized trials demonstrating superiority. Yet surveillance protocols have proliferated through institutional habit and defensive reasoning, immune to the same scrutiny. The result is a peculiar burden of proof: proponents of de-escalation must demonstrate non-inferiority, while proponents of intensive monitoring face no reciprocal obligation. This is not science; it is tradition masquerading as prudence.

The broader oncology literature offers little comfort to surveillance maximalists. A Cochrane systematic review of 53 trials involving over 20,000 cancer survivors found that less intensive follow-up made little or no difference to overall survival [2]. The assumption that earlier detection improves outcomes presupposes effective salvage treatments and that lead-time changes the disease course. For cT1 RCC treated with ablation, where cancer-specific survival exceeds 95%, these presuppositions require justification. Dabestani et al demonstrated in a European multicenter database that increased cross-sectional imaging frequency failed to improve post-recurrence survival in surgically treated localized RCC [3]. Detection timing, it appears, matters less than tumor biology.

The harms of intensive surveillance, by contrast, are not hypothetical. Smith-Bindman and colleagues projected real lifetime cancer risks from repeated CT examinations [4]. For younger patients and those with hereditary RCC syndromes requiring potentially lifelong monitoring, cumulative radiation exposure becomes a real harm weighed against an unquantified benefit. Singer et al documented poor adherence to CT referral guidelines across European centers [5], suggesting we have normalized radiation exposure. Post-ablation surveillance is one area where we could reduce exposure.

Beyond radiation, the psychological toll of repeated imaging also matters. “Scanxiety” is well-documented in cancer survivors and may paradoxically diminish quality of life in patients with excellent prognoses [6]. Wullaert et al found that reduced surveillance intensity did not compromise health-related quality of life and occasionally improved psychological well-being [7]. Each scan we order is not merely a diagnostic test but an intervention with its own risk-benefit profile, including the anxiety it generates. The economic dimension compounds these concerns: Lobo et al demonstrated that intensive protocols incur substantially higher costs without proportionate improvements in recurrence detection [8].

The limitations of this review are real. Retrospective data and moderate-to-high risk of bias preclude causal conclusions. No studies adhered to the 2024 EAU recommendations, meaning reduced surveillance remains a reasonable hypothesis rather than a validated practice. The comparison between imaging strategies cannot account for confounders, including tumor characteristics and institutional expertise. These limitations, however, apply equally to the evidence supporting current intensive practice, which is to say: there is none.

What should clinicians do with this uncertainty? Three recommendations follow. First, distinguish clearly between early post-ablation imaging to confirm technical success (typically at 1–3 months) and long-term oncological surveillance. These serve different purposes and should not be conflated in follow-up protocols. Second, apply the 2024 EAU recommendation to consider terminating surveillance for low-risk patients (cT1a, low-grade, complete ablation) after 3 years, particularly in older patients with competing mortality risks [9]. Third, engage patients in shared decision-making about surveillance intensity, presenting honestly that the benefits of frequent imaging are assumed rather than proven, while the harms of radiation exposure, anxiety, and cost are real.

Future research must address this evidence gap directly. Randomized trials comparing guideline-recommended frequencies against current practice are essential, though challenging to conduct given the long follow-up required and low event rates. Risk-stratification tools incorporating tumor biology and patient factors could allow personalized surveillance. Emerging biomarkers such as urinary glycosaminoglycans offer potential non-invasive alternatives [10]. Until we have such data, we should acknowledge that our current practice represents a collective choice to prioritize action over evidence.

The study by Reijerink and colleagues will not resolve the surveillance debate. Its value lies in making visible what we have chosen not to see: that intensive post-ablation imaging is an intervention we have adopted without the evidence we would require of any other medical practice. The burden of proof has been assigned backward. Those advocating restraint are asked to demonstrate safety, while those ordering scans face no obligation to demonstrate benefit. Reversing this asymmetry would not mean abandoning surveillance but subjecting it to the same evidentiary standards we apply elsewhere. In medicine, the absence of evidence of harm has never been a sufficient justification for intervention. Why should surveillance be different?

## References

[1] Reijerink MAA, van den Brink L, Henderickx MMEL et al (2025) The use of computed tomography during follow-up after ablation of cT1 renal cell carcinoma: evidence for overuse. Eur Radiol. 10.1007/s00330-026-12345-6 10.1007/s00330-026-12345-6 PMC13282219 41649536

[2] Høeg BL, Bidstrup PE, Karlsen RV et al (2019) Follow-up strategies following completion of primary cancer treatment in adult cancer survivors. Cochrane Database Syst Rev 2019:CD012425. 10.1002/14651858.CD012425.pub2 31750936 10.1002/14651858.CD012425.pub2 PMC6870787

[3] Dabestani S, Beisland C, Stewart GD et al (2019) Increased use of cross-sectional imaging for follow-up does not improve post-recurrence survival of surgically treated initially localized RCC: results from a European multicenter database (RECUR). Scand J Urol 53:14–20. 10.1080/21681805.2019.1588919 30907214 10.1080/21681805.2019.1588919

[4] Smith-Bindman R, Chu PW, Azman Firdaus H et al (2025) Projected lifetime cancer risks from current computed tomography imaging. JAMA Intern Med 185:710–719. 10.1001/jamainternmed.2025.0505 40227719 10.1001/jamainternmed.2025.0505 PMC11997853

[5] Singer C, Saban M, Luxenburg O et al (2025) Computed tomography referral guidelines adherence in Europe: insights from a seven-country audit. Eur Radiol 35:1166–1177. 10.1007/s00330-024-11083-x 39384590 10.1007/s00330-024-11083-x PMC11835886

[6] Rossi SH, Klatte T, Stewart GD (2018) Quality of life outcomes in patients with localised renal cancer: a literature review. World J Urol 36:1961–1972. 10.1007/s00345-018-2415-3 30051264 10.1007/s00345-018-2415-3 PMC6280814

[7] Wullaert L, Voigt KR, Verhoef C, Husson O, Grunhagen DJ (2023) Oncological surgery follow-up and quality of life: meta-analysis. Br J Surg 110:655–665. 10.1093/bjs/znad022 36781387 10.1093/bjs/znad022 PMC10364539

[8] Lobo JM, Nelson M, Nandanan N, Krupski TL (2016) Comparison of renal cell carcinoma surveillance guidelines: competing trade-offs. J Urol 195:1664–1670. 10.1016/j.juro.2015.12.094 26778713 10.1016/j.juro.2015.12.094

[9] Ljungberg B, Albiges L, Abu-Ghanem Y et al (2022) European Association of Urology guidelines on renal cell carcinoma: the 2022 update. Eur Urol 82:399–410. 10.1016/j.eururo.2022.03.006 35346519 10.1016/j.eururo.2022.03.006

[10] van den Brink L, Reijerink MAA, Henderickx MMEL et al (2024) Is frequent imaging necessary? Impact of computed tomography during follow-up after surgical treatment for nonmetastatic renal cell carcinoma: a systematic review. Eur Urol Oncol 8:829–840. 10.1016/j.euo.2024.11.014 39665918 10.1016/j.euo.2024.11.014

